# BRG1 governs glucocorticoid receptor interactions with chromatin and pioneer factors across the genome

**DOI:** 10.7554/eLife.35073

**Published:** 2018-05-24

**Authors:** Jackson A Hoffman, Kevin W Trotter, James M Ward, Trevor K Archer

**Affiliations:** 1 Epigenetics and Stem Cell Biology LaboratoryNational Institute of Environmental Health Sciences, National Institutes of HealthNorth CarolinaUnited States; 2 Integrative BioinformaticsNational Institute of Environmental Health Sciences, National Institutes of HealthNorth CarolinaUnited States; Stowers Institute for Medical ResearchUnited States

**Keywords:** chromatin, transcription factors, brg1, glucocorticoid receptor, pioneer factors, histone modifications, Human

## Abstract

The Glucocorticoid Receptor (GR) alters transcriptional activity in response to hormones by interacting with chromatin at GR binding sites (GBSs) throughout the genome. Our work in human breast cancer cells identifies three classes of GBSs with distinct epigenetic characteristics and reveals that BRG1 interacts with GBSs prior to hormone exposure. The GBSs pre-occupied by BRG1 are more accessible and transcriptionally active than other GBSs. BRG1 is required for a proper and robust transcriptional hormone response and knockdown of BRG1 blocks recruitment of the pioneer factors FOXA1 and GATA3 to GBSs. Finally, GR interaction with FOXA1 and GATA3 binding sites was restricted to sites pre-bound by BRG1. These findings demonstrate that BRG1 establishes specialized chromatin environments that define multiple classes of GBS. This in turn predicts that GR and other transcriptional activators function via multiple distinct chromatin-based mechanisms to modulate the transcriptional response.

## eLife digest

Steroid hormones play a number of roles in the body, including controlling the immune system and the body’s response to stress. Artificially produced steroid hormones may also be used as part of treatments for cancer. The hormones affect the behavior of cells by binding to and activating hormone receptor proteins. The receptors can then interact with the cell’s DNA to change the activity of nearby genes.

Gaining access to particular sites on a strand of DNA is not always easy. Cells pack DNA into a structure called chromatin. In some regions the DNA is so tightly wrapped in the chromatin that the receptors cannot access it. The structure of the chromatin therefore affects how a cell responds to steroid hormones.

Inaccessible regions of chromatin can be ‘opened up’ by two groups of proteins, known as remodeling proteins and pioneer factors. Hormone receptors can work with these proteins to access particular DNA regions, but exactly how all these proteins work together was not fully understood.

Hoffman et al. have now used DNA and RNA sequencing technologies to examine the roles of a hormone receptor called the glucocorticoid receptor, a remodeling protein called BRG1, and various pioneer factors in human breast cancer cells. This revealed three ways in which the glucocorticoid receptors worked with the other proteins when binding to chromatin. These could be distinguished by the pattern of BRG1 molecules bound to the DNA.

Further investigation showed that BRG1 controls how the glucocorticoid receptor affects the activity of genes. In addition, BRG1 influences how the receptor interacts with pioneer factors when it is bound to DNA. Future research into how these proteins work together could ultimately help us to improve how we use steroid hormones to treat diseases.

## Introduction

The Glucocorticoid Receptor (GR, encoded by the NR3C1 gene) is a type I nuclear receptor that elicits the transcriptional response to glucocorticoid steroid hormones. This transcriptional response is essential for human health and development. Glucocorticoids such the synthetic hormone Dexamethasone (Dex) are utilized to activate GR signaling to treat human auto-immune and inflammatory diseases and to promote fetal lung development. Understanding the mechanisms through which the transcriptional response to glucocorticoids is generated is critical for human health and for the further development of disease treatments. A detailed mechanism for GR transcriptional activity has been established through the examination of GR at model genes such as the Mouse Mammary Tumor Virus (MMTV). Upon hormone binding, GR enters the nucleus and binds to regions in the chromatin known as GR binding sites (GBSs). At MMTV, GR binding triggers the recruitment of other factors including the SWI/SNF chromatin remodeling complex (Cordingley et al., 1987; Fryer and Archer, 1998). Recruitment of the SWI/SNF complex induces the reorganization of nucleosomes around GR binding sites, which in turn facilitates binding of other transcription factors and potentiates transcriptional activation (Archer et al., 1994; Wallberg et al., 2000).

The mammalian SWI/SNF chromatin remodeling complex is comprised of one of two catalytic ATPases, BRG1 and BRM, and 10 or more BRM/BRG1-asssociated factor (BAF) subunits. The so-called BAF complex is critical throughout embryonic development and is among the most commonly mutated protein complexes in human cancers (Shain and Pollack, 2013; Kadoch et al., 2013; Wu et al., 2017). BRG1 and the BAF subunits also play critical roles in mediating the transcriptional response to glucocorticoid signaling. GR interacts directly with BAF57, BAF60A, and BAF250, and requires the catalytic ATPase activity of BRG1 to promote transcriptional activation of MMTV (Nie et al., 2000; Inoue et al., 2002; Hsiao et al., 2003). Chromatin remodeling by the BAF complex was required for the subsequent recruitment of RNA Polymerase II and other transcription factors to the MMTV promoter (Johnson et al., 2008). Transcriptional activation of MMTV was also promoted by the recruitment of a complex containing Ku70/86, Topoisomerase IIβ, and Poly(ADP-ribose) polymerase one by BRG1 (Trotter et al., 2015). Thus, chromatin remodeling through the BAF complex is a critical component of GR signaling.

Beyond the requirement for chromatin remodeling by the BAF complex, the underlying chromatin landscape appears to play a crucial role in patterning the hormone response. GR preferentially binds to regions in the chromatin that are pre-accessible as measured by DNase hypersensitivity or formaldehyde-assisted isolation of regulatory elements (John et al., 2011; Burd et al., 2012). These findings indicated that GR chromatin interactions were predetermined by other chromatin interacting factors. Pioneer factors, transcription factors that can bind to and open regions of closed chromatin, have been implicated in the pre-patterning of GR binding. The pioneer factors C/EBPβ and AP1 pre-occupied a large proportion of GR binding sites in mouse liver and mammary cells, and were required to maintain chromatin accessibility at GR binding sites (Biddie et al., 2011; Grøntved et al., 2013). Similarly, FOXA1 pre-bound a large number of Estrogen Receptor (ER, another nuclear hormone receptor closely related to GR) binding sites and was required for ER binding and transcriptional activity. Conversely, recent work has demonstrated that hormone signaling through both ER and GR promoted the redistribution of FOXA1 chromatin interactions (Swinstead et al., 2016). These findings helped to demonstrate that current models of GR activity fail to fully account for the complexities of GR signaling at a genomic scale, and that more sophisticated and diverse models are required to describe the mechanisms through which GR initiates a transcriptional response.

In this study, we examined mechanisms of GR transcriptional regulation through genome-scale analyses of hormone-induced changes in transcriptional activity and the binding patterns of GR, BRG1, and pioneer factors. We identify distinct classes of GR binding site based upon the binding profile of BRG1 before and after hormone treatment. BRG1 binding to GR sites prior to hormone marked GR binding sites that were pre-accessible and enriched for marks of transcriptionally active chromatin. BRG1 was required for a robust GR transcriptional response, as disruption of BRG1 expression dramatically altered the profile of hormone-induced differentially expressed genes. GR binding sites that were pre-bound by BRG1 were also enriched for motifs of pioneer factors such as FOXA1 and GATA3, and BRG1 binding at pioneer factor binding sites in untreated cells was predictive of GR binding upon hormone treatment. Furthermore, BRG1 expression was required for Dex-induced recruitment of additional FOXA1 and GATA3 to GR binding sites. Taken together, our data suggest that GR elicits the transcriptional response to hormone via multiple distinct mechanisms that are reliant on the pre-patterning of specialized chromatin environments through the actions of the BAF complex and additional factors.

## Results

### Differential patterns of BRG1 interaction define three classes of GR binding site

Current models of GR function commonly depict the hormone-dependent recruitment of the BRG1-containing SWI/SNF chromatin remodeling complex to GBSs (Cordingley et al., 1987; Fryer and Archer, 1998; Archer et al., 1994; Wallberg et al., 2000). This recruitment of BRG1 facilitates the opening of chromatin around the GBS to enhance the ability of GR to elicit transcriptional effects. However, recent work demonstrates that GBSs exhibit chromatin accessibility prior to hormone treatment, suggesting that some mechanism pre-patterns the chromatin environment around GBSs (John et al., 2011; Burd et al., 2012).

**Figure 1.**
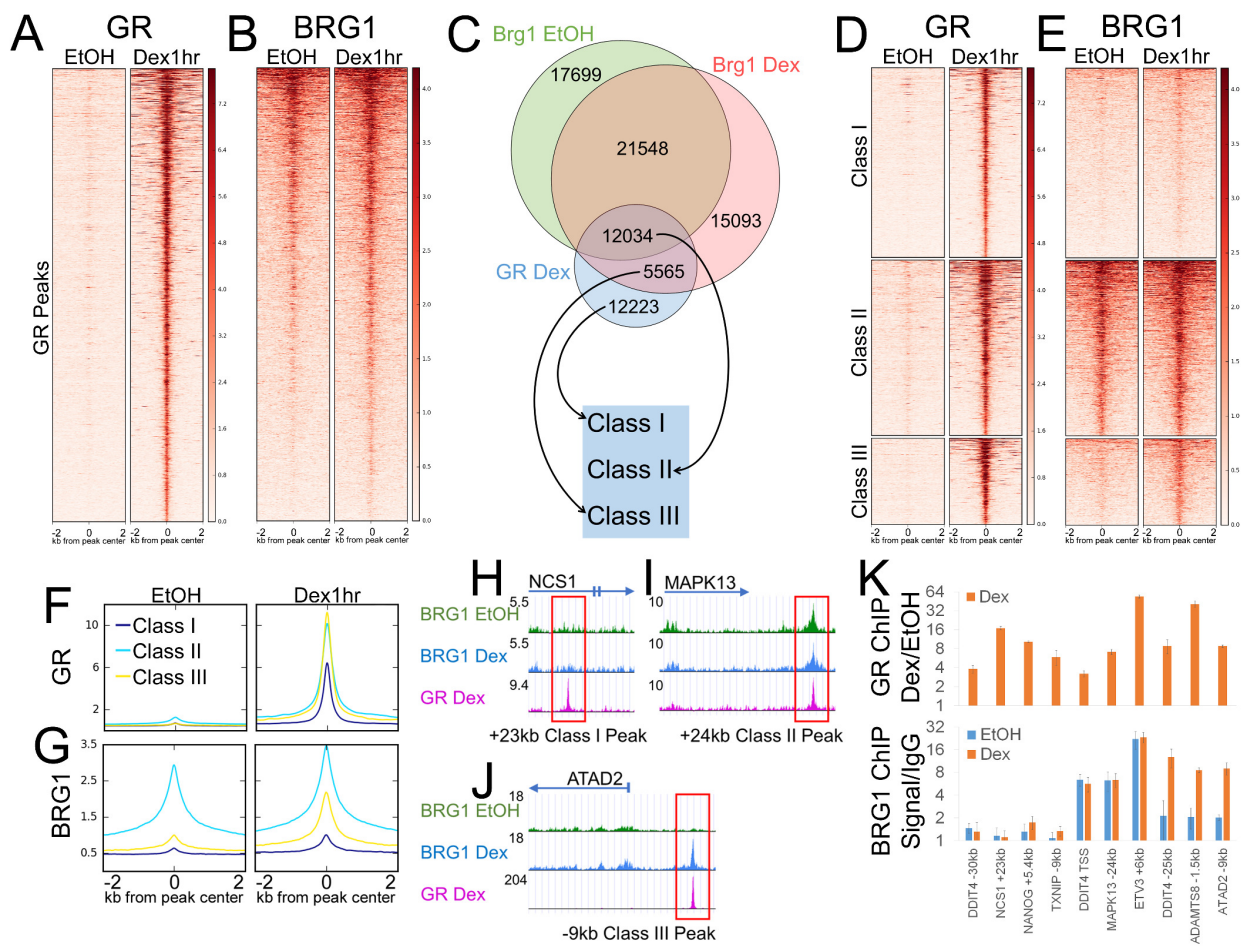
BRG1 chromatin interaction defines distinct classes of GR binding site. (**A**) Heatmap demonstrating that GR is detected at 29934 binding sites following 1 hr Dex treatment. (**B**) Heatmaps illustrating detection of BRG1 at a subset of GR binding sites prior to hormone treatment and recruitment of BRG1 to additional sites following 1 hr of Dex treatment. (**C**) Venn diagram of overlap between GR, BRG1-EtOH, and BRG1-Dex peaks and the designation of three classes of GR peaks. (**D**) Heatmap of GR signal over GR peaks divided into three classes. (**E**) Heatmap of BRG1 signal over GR peaks divided into three classes. (**F–G**) Meta-profiles of GR and BRG1 ChIP-seq coverage over GR peak classes. (**H–J**) UCSC genome browser snapshots of GR, BRG1 EtOH, and BRG1 Dex ChIP-seq coverage at representative Class I, II, and III GR peaks. (**K**) ChIP-QPCR validation of GR and BRG1 ChIP enrichment at representative Class I, II, and III GR peaks.

To investigate a potential role for BRG1 in this phenomenon, we preformed chromatin immunoprecipitation with high-throughput sequencing (ChIP-seq) in the A1-2 model cell line (Figure 1) (Archer et al., 1994). We obtained data of high depth (>60 million reads per GR or BRG1 ChIP-seq) and called peaks using a 0.001 false discovery rate cutoff to ensure high confidence in identifying GR and BRG1 binding sites. One hour of hormone exposure was sufficient to induce a massive DNA binding response by GR, with 29934 GR binding sites/peaks identified specifically in Dex-treated cells (Figure 1A). The number of peaks called from our dataset falls within the range of peak numbers called by GR ChIP-seq experiments in other cell lines (Swinstead et al., 2016; Starick et al., 2015). Dex treatment also had a robust effect on the chromatin localization of BRG1. While 50,000 + BRG1 peaks were identified in each condition, only 33582 peaks were shared while 17699 were specific to EtOH-treated cells and 20658 were specific to Dex-treated cells (Figure 1C, Figure 1—figure supplement 1A). This rearrangement of BRG1 chromatin localization is consistent with BRG1 being recruited to GBSs upon hormone treatment. To verify this, we examined the overlap between BRG1 and GR peaks and found that 58% of GR peaks are overlapped by a BRG1 peak (Figure 1B,C). Surprisingly, there was significant overlap in both EtOH and Dex-treated cells (Figure 1B), with 12034 GR peaks bound by BRG1 in both conditions, and 5565 bound by BRG1 in a Dex-specific manner (Figure 1C). 12223 GR peaks were not bound by BRG1, and a total of 54340 BRG1 peaks (including 15093 Dex-specific and 17699 EtOH-specific) did not overlap GR (Figure 1C, Figure 1—figure supplement 1B). These findings indicate that a large subset of the subsequent GR peaks are bound by BRG1 prior to hormone treatment consistent with the concept that BRG1 could be involved in pre-patterning GBSs.

**Figure 1—figure supplement 1.**
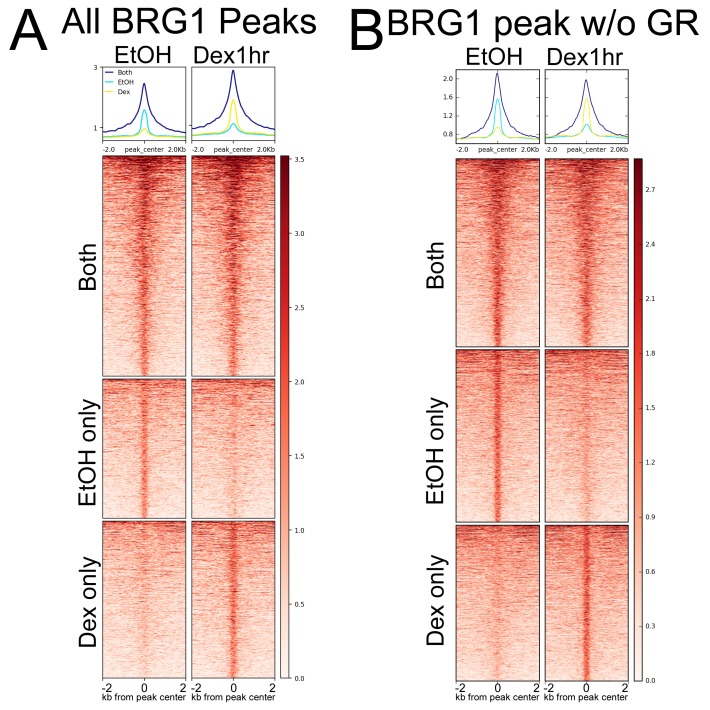
Hormone treatment reorganizes BRG1 chromatin localization. (**A**) Heatmap depicting BRG1 ChIP-seq coverage at BRG1 peaks that are called in EtOH only, Dex only, or both conditions. (**B**) Heatmap depicting BRG1 ChIP-seq coverage at BRG1 peaks as in (**A**), but with peaks that overlap GR peaks removed (12034 Class II and 5565 Class III peaks removed).

**Figure 1—figure supplement 2.**
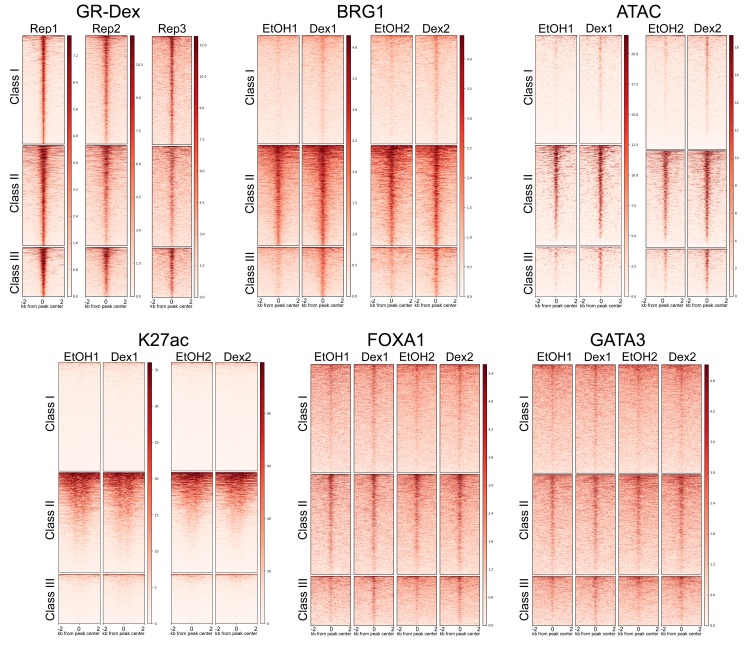
Biological replication of Next Generation Sequencing data sets. Heatmaps depicting consistency between biological replicate ChIP-seq and ATAC-seq experiments over GR peak classes

To further dissect the relationship between GR and BRG1, we defined three classes of GR peak: Class I peaks as GR peaks lacking any overlap with a BRG1 peak, Class II peaks as GR peaks overlapped by BRG1 peaks in both EtOH- and Dex-treated conditions, and Class III peaks as GR peaks that overlapped only Dex-specific BRG1 peaks (Figure 1C). Collectively, Class I GR peaks were narrower and showed less overall GR enrichment than Class II or Class III peaks (Figure 1D,F). BRG1 was not enriched at Class I GR peaks, was constitutively enriched at Class II GR peaks, and was induced by Dex-treatment at Class III peaks (Figure 1E,G). The peak classes were easily identifiable at gene level coverage (Figure 1H–J) and GR and BRG1 enrichment patterns were independently validated by ChIP-QPCR (Figure 1K). Thus, we utilized these three GR peak classes in our subsequent analyses to examine how differential patterns of BRG1 interaction could define the GR-mediated hormone response.

### Class II GR peaks are associated with open and transcriptionally active chromatin

**Figure 2.**
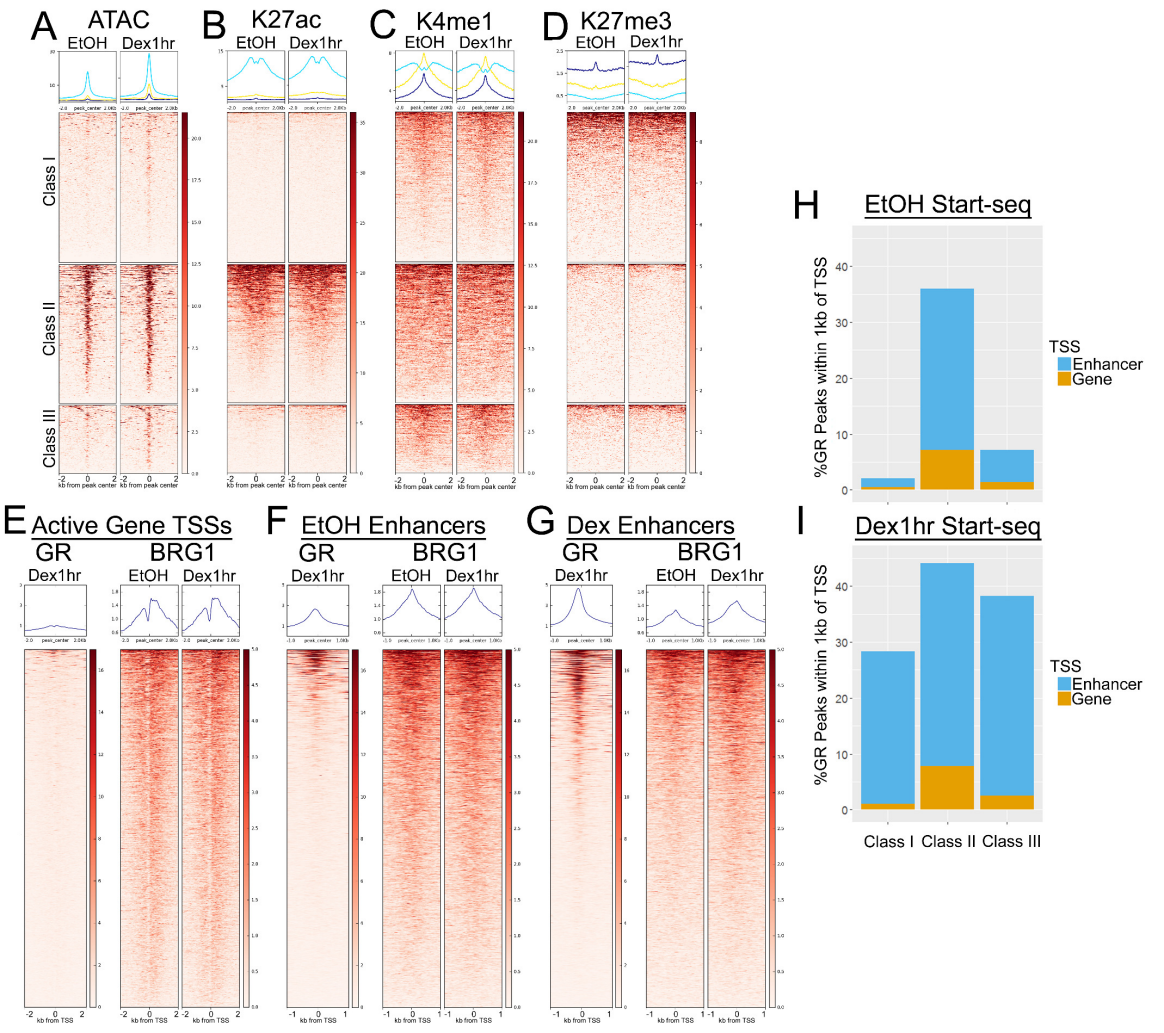
Class II GR peaks are associated with open and transcriptionally active chromatin. (**A**) ATAC-seq accessibility/short reads are enriched over Class II GR peaks independently of hormone treatment. (**B**) H3K27ac ChIP-seq coverage is specifically enriched over Class II GR peaks independently of hormone treatment. (**C**) H3K4me1 ChIP-seq is enriched over Class I and III peaks, whereas Class II peaks have a broader enrichment pattern with a trough directly over the GR peak. (**D**) H3K27me3 is not strongly enriched at any of the GR peak classes. (**E–G**) Heatmaps depicting GR and BRG1 ChIP-seq signal around active gene TSSs (**E**), enhancer TSSs from EtOH/untreated cells (**F**), and enhancer TSSs from Dex 1 hr cells (**G**). (**H–I**) Stacked barplots showing the percentage of GR peaks that are within 1 kb of an active gene TSSs (orange) or enhancer TSSs (blue) in using Start-seq TSS calls from untreated cells (**H**) and Dex 1 hr cells (**I**).

Given the differences in BRG1 distribution we next sought to determine whether the GR peak classes also exhibited distinct chromatin environments. Short, sub-nucleosome length ATAC-seq reads were used as a measure of chromatin accessibility, and were strongly enriched at Class II peaks independent of Dex treatment. Thus, Class II GR peaks are accessible prior to hormone treatment (Figure 2A). Conversely, minimal accessibility was detected at Class I and III GR peaks in EtOH-treated cells, indicating that prior to hormone treatment, these GR peaks were largely inaccessible (Figure 2A). Upon 1 hr Dex exposure, low but distinct levels of accessibility were induced, predominantly at class III peaks where BRG1 binding was also induced (Figure 2A). Thus, chromatin accessibility at GR peak classes was directly correlated with the pattern of BRG1 occupancy.

**Figure 2—figure supplement 1.**
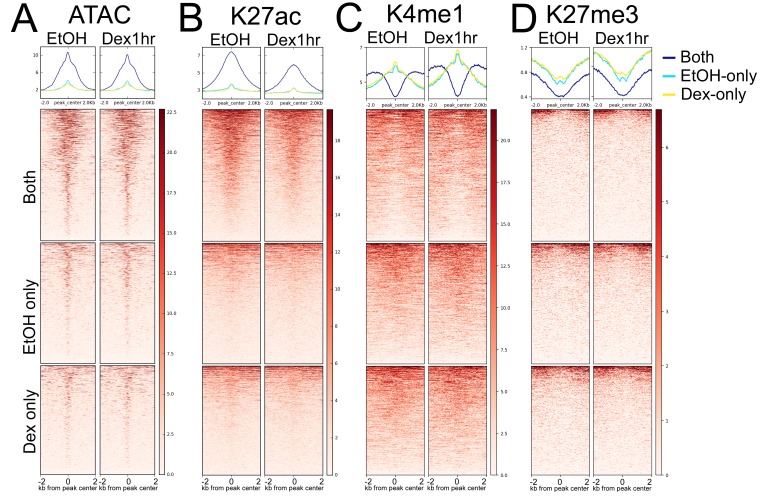
BRG1 peaks without GR are accessible and have active chromatin marks. BRG1 peaks without GR that are hormone-independent (labeled ‘Both’) have enrichment patterns for ATAC accessibility, H3K27ac, and H3K4me1 that are similar to Class II peaks. EtOH-specific and Dex-specific BRG1 peaks have patterns more similar to Class I or III peaks.

**Figure 2—figure supplement 2.**
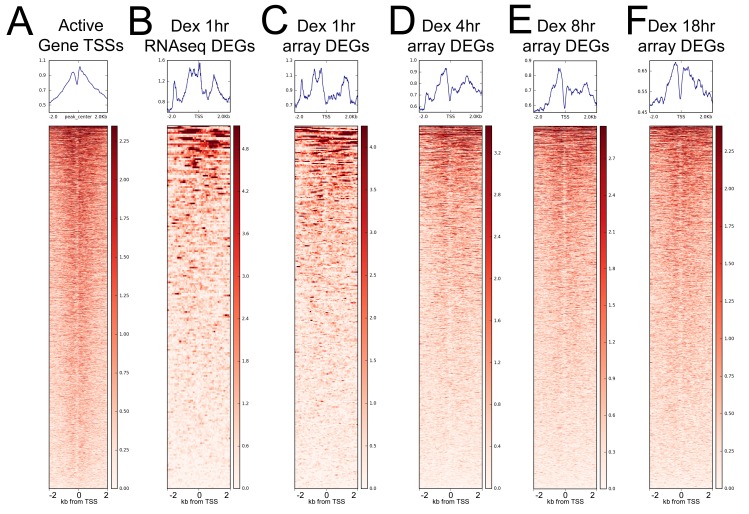
GR is weakly enriched around TSSs. Heatmaps showing GR ChIP-seq coverage at (**A**) active gene TSSs (same plot as Figure 2e with adjusted y and z scales), (**B**) TSSs of RNA-seq identified DEGs following 1 hr Dex treatment, (**C**) TSSs of microarray-identified DEGs following 1 hr Dex treatment, (**D**) TSSs of microarray-identified DEGs following 4 hr Dex treatment, (**E**) TSSs of microarray-identified DEGs following 8 hr Dex treatment, and (**F**) TSSs of microarray-identified DEGs following 18 hr Dex treatment.

**Figure 2—figure supplement 3.**
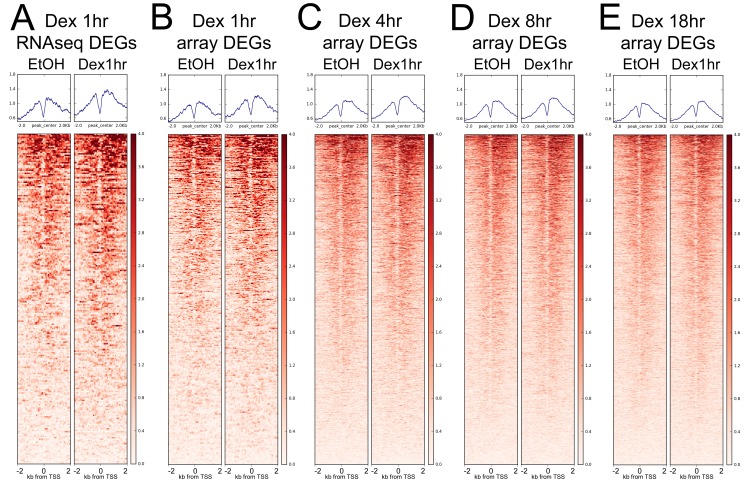
BRG1 is enriched around TSSs. Heatmaps showing BRG1 +/- Dex at ChIP-seq coverage at (**A**) TSSs of RNA-seq identified DEGs following 1 hr Dex treatment, (**B**) TSSs of microarray-identified DEGs following 1 hr Dex treatment, (**C**) TSSs of microarray-identified DEGs following 4 hr Dex treatment, (**D**) TSSs of microarray-identified DEGs following 8 hr Dex treatment, and (**E**) TSSs of microarray-identified DEGs following 18hr Dex treatment.

We next examined histone modifications at the GR peak classes for differentially enriched active or repressive marks. Histone 3 Lysine 27 acetylation (K27ac), a histone modification associated with transcriptionally active chromatin at TSSs and active enhancers, was only detected at Class II GR peaks (Figure 2B). Histone 3 Lysine four monomethylation (K4me1), a marker of both inactive/poised and active enhancers, was enriched at all three peaks classes, but had a unique pattern at Class II peaks (Figure 2C). While K4me1 enrichment was centered on the GR peak at Class I and III peaks, Class II peaks had broader K4me1 enrichment with an apparent trough of enrichment directly over the GR peak (Figure 2C). None of the peak Classes displayed strong enrichment of Histone 3 Lysine 27 trimethylation (K27me3), a repressive chromatin modification (Figure 2D). Taken together with the ATAC-seq data, the patterns of histone modifications at GR peaks were associated with enhancer-like chromatin marks. The strong ATAC accessibility signal and K27ac enrichment at Class II peaks suggested that they might represent GR binding to active enhancers. On the other hand, the relatively low levels of ATAC accessibility and the patterns of K4me1 and K27ac at Class I and III peaks suggested that they represented GR binding to inactive or poised enhancers. Furthermore, Class II GR peaks represent a distinct set of GR peaks that are associated with BRG1 as well as marks of accessible and transcriptionally active chromatin.

To characterize transcriptional events associated with GR chromatin interaction, we investigated whether any GR peaks were proximal to functionally engaged transcriptional start sites (TSSs). We previously used Start-seq to identify actively transcribed TSSs in A1-2 cells (Lavender et al., 2016). We divided these TSSs into those correlated with active, annotated gene TSSs and those that were greater than 2 kb from any gene TSS and represent putative active enhancer TSSs. GR ChIP-seq signal after 1 hr of Dex treatment was modestly enriched over active gene TSSs (Figure 2—figure supplement 2), however this was dwarfed by the level of GR enrichment at active enhancer TSSs identified in either EtOH- and Dex-treated cells (Figure 2E–G). The average GR enrichment over active gene TSSs was not markedly increased when the analysis was restricted to genes that were differentially expressed (DEGs) following 1, 4, 8, or 18 hr of Dex treatment (Figure 2—figure supplement 2) Thus, GR was much more frequently associated with enhancer transcription than gene transcription. Unlike GR, BRG1 ChIP-seq signal was broadly detected and similarly enriched at all active TSSs (Figure 2E). Over active gene TSSs and EtOH-detected active enhancers, the average levels BRG1 enrichment appeared unaffected by Dex (Figure 2E–F). This was consistent with the predominantly Class II-specific enrichment of ATAC-seq and H3K27ac ChIP-seq signal and indicated that BRG1 was constitutively associated with most active TSSs in A1-2 cells. Furthermore, ATAC-seq accessibility and K27ac were also strongly enriched at hormone-independent BRG1 peaks that did not overlap GR peaks (Figure 2—figure supplement 1). Taken together, these data indicated that BRG1 was largely associated with open and active chromatin independently of GR, and that BRG1 peaks that were not affected by hormone treatment most strongly exhibited these characteristics. However, a Dex-induced increase in BRG1 enrichment was observed at active enhancer TSSs detected in Dex-treated cells (Figure 2G), and more modestly at the TSSs of Dex-induced DEGs (Figure 2—figure supplement 3). Thus, TSSs with altered transcriptional activity upon Dex exposure also exhibited hormone-induced BRG1 enrichment.

K27ac and K4me1 ChIP-seq suggested that GR peak classes were differentially associated with active and inactive/poised enhancers (Figure 2B–C). To further dissect this relationship, we looked to see how many peaks in each class were in close proximity to active enhancer and gene TSSs. When considering active TSSs called in untreated cells, 38% of Class II peaks were within 1 kb of a TSS, compared to 2% of Class I peaks and 7% of Class III peaks (Figure 2H). However, when considering active TSSs called in cells treated with Dex for 1 hr, a significant portion of each peak class was within 1 kb of a TSS (26% Class I, 45% Class II, and 38% Class III) (Figure 2I). Despite the induction of transcriptional activity near a significant subset of Class I and III peaks, K27ac was not observably induced at these peaks (Figure 2B) whereas the pattern of K4me1 was unaffected (Figure 2C). Taken together with the patterns of K27ac and K4me1, these data indicated that Class II peaks represented GR interactions with accessible chromatin and transcriptionally active enhancers. Furthermore, they indicated that Class I and III peaks represented GR interactions with inactive or poised enhancers that could be activated upon GR binding, but exhibited limited accessibility and were devoid of the K27ac mark.

### BRG1 is required for the transcriptional hormone response

**Figure 3.**
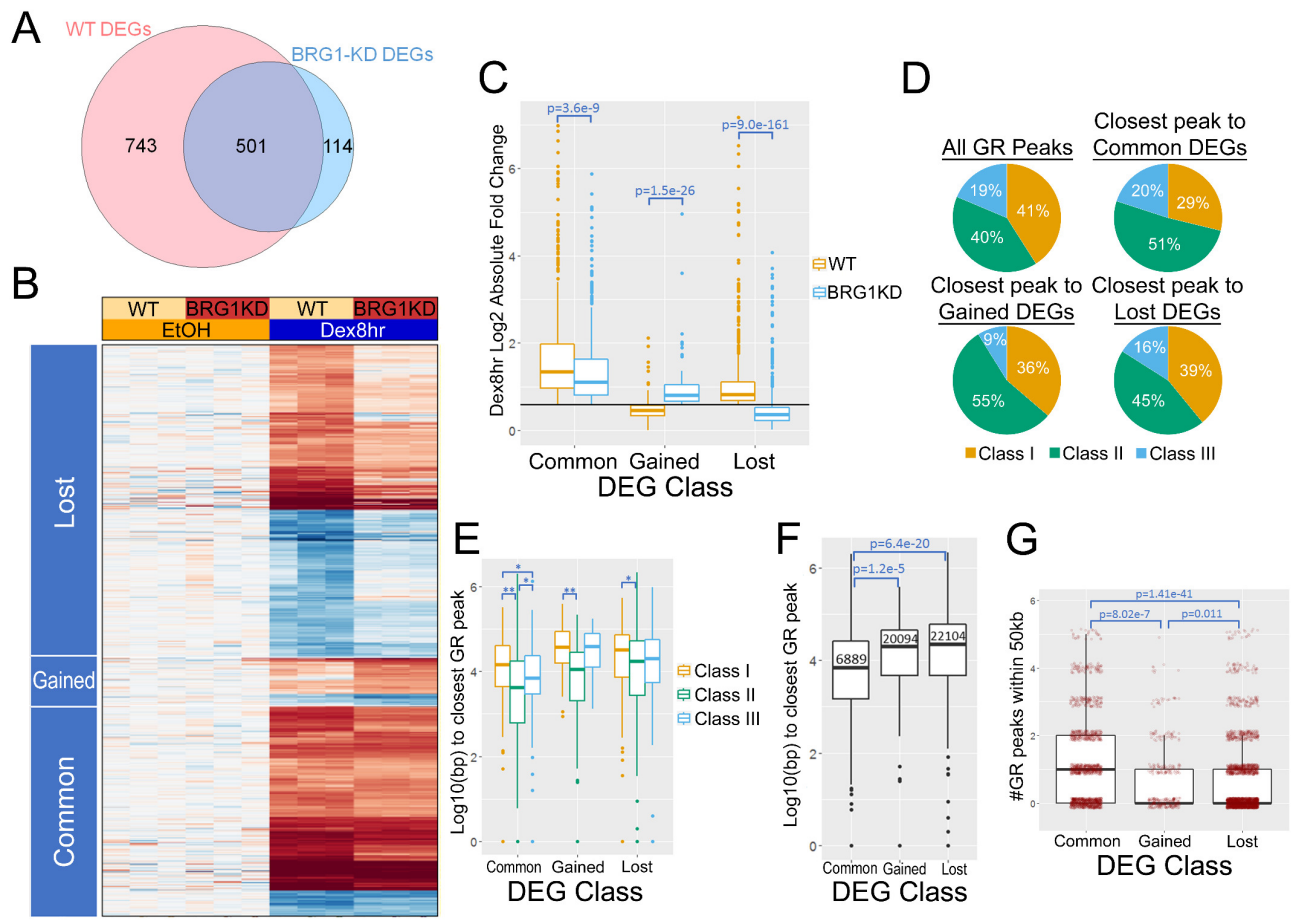
BRG1 is required for the Dex-induced transcriptional response. (**A**) Venn diagram showing overlap between DEGs in 8 hr Dex treatment vs EtOH in WT and BRG1-KD cells. (**B**) Heatmap depicting log2 fold change of DEGs in 8 hr Dex treatment vs EtOH in WT and BRG1-KD cells. (**C**) Box plots of log two absolute fold changes of 8 hr Dex DEGs. Black line depicts 1.5 fold change. (**D**) Pie charts depicting the class of the closest GR peak to each DEG TSS. (**E**) Box plots depicting the log 10 distance from the DEG TSSs to their closest GR peaks divided into GR peak classes. *=p value<0.05, **=p value<0.001 (**F**) Box plots showing the distance from DEG TSSs to their closest GR peaks. Median distances are labeled in base pairs. (**G**) Box and scatter plots depicting the number of GR peaks within 50 kb of the TSS of 8 hr Dex DEGs. Outlier peaks with more than 5 GR peaks within 50 kb of the TSS were omitted from the graph for display purposes. All p-values were calculated using Wilcoxon rank sum tests.

To further investigate the role of BRG1 in regulating the transcriptional hormone response, we performed RNA-seq in cells which harbor an inducible shRNA targeting BRG1 (A1A3 cells, previously described in [Burd et al., 2012]). Treatment with Doxycycline for 72 hr resulted in an 80–85% reduction in BRG1 protein levels as well as partial reduction in the nuclear levels of GR protein (Figure 3—figure supplement 1A). RNA-seq performed at 1 hr Dex treatment in A1-2 cells yielded approximately 200 DEGs (Lavender et al., 2016). In order to capture a more robust transcriptional hormone response for analysis, we used an 8 hr Dex treatment in A1A3 cells. In normal conditions, 1244 DEGs (Fold Change > 1.5, p-value<0.01, false discovery rate <0.05) were identified following 8 hr of Dex treatment (Figure 3A). 743 of these DEGs failed to meet the same fold-change and significance cutoffs in Dex treated BRG1-KD cells, indicating BRG1 was required the transcriptional response to Dex. Intriguingly, BRG1-KD cells had 114 Dex-regulated DEGs that were not called DEGs in control Dex-treated cells, indicating that BRG1 also suppressed the hormone responsiveness of a small number of genes (Figure 3A). Visualizing the changes in gene expression by heatmap revealed that while the hormone response was largely muted or suppressed following BRG1 knockdown, a significant number of genes showed equal or greater hormone responses following BRG1 knockdown (Figure 3B). Indeed, the absolute fold change of both ‘common’ and ‘lost’ DEGs was reduced in BRG1-KD cells, and increased in ‘gained’ DEGs (Figure 3C). Together, these data indicated that BRG1 was required for both a robust transcription hormone response and to suppress ectopic hormone responsiveness.

**Figure 3—figure supplement 1.**
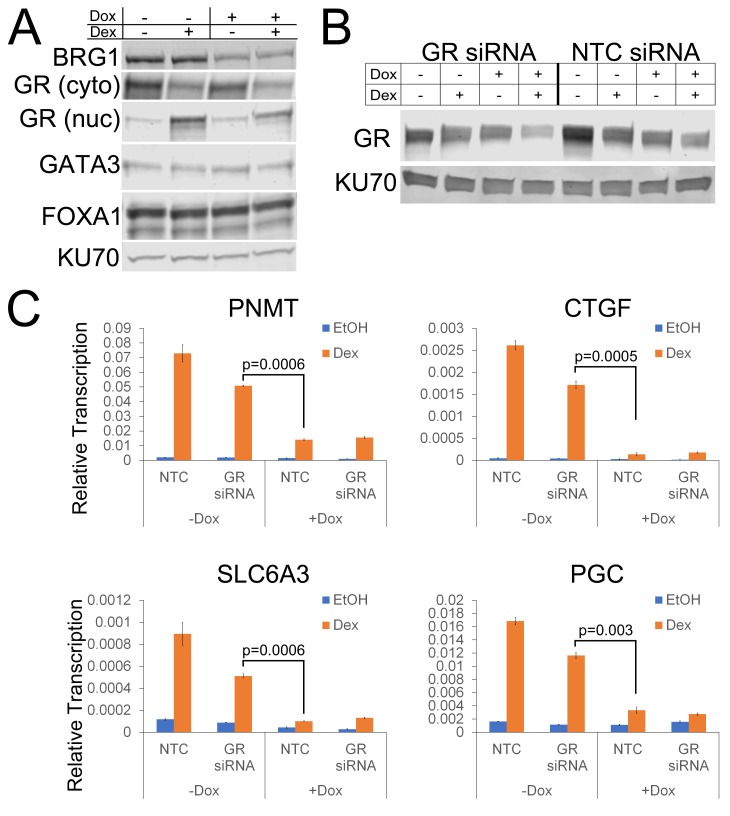
BRG1 and GR knockdown in A1A3 cells. (**A**) Western blots depicting protein levels of BRG1, cytoplasmic GR, nuclear GR, GATA3, FOXA1, and KU70 in A1A3 cells following BRG1 knockdown by doxycycline treatment. (**B**) Western blots depicting protein levels of GR and KU70 following transfection with GR siRNA and/or BRG1 knockdown by doxycycline treatment. (**C**) QPCR demonstrating the effects of GR siRNA and BRG1 knockdown on ‘lost’ target genes. Note that while GR siRNA partially reduces dex-dependent induction of transcription, BRG1 knockdown results in a greater loss of dex-dependent induction.

Decreased levels of GR protein in BRG1-KD cells suggested that part of the BRG1 effect might instead result from insufficient nuclear GR. While the increased hormone-responsiveness of the 114 ‘gained’ DEGs in BRG1-KD cells served as evidence that this was not the case, we sought to directly test whether decreased GR was the dominant factor in the loss of dex-induction of ‘lost’ DEGs. Reduction of GR protein levels by 50–70% by siRNA resulted in a modest loss of dex-induced transcription at candidate ‘lost’ genes (Figure 3—figure supplement 1B–C). However, BRG1-KD resulted in a much stronger loss of dex-induced transcription (Figure 3—figure supplement 1C). As such, these data strongly suggest that the changes in the Dex response in A1A3 cells are predominantly driven by the silencing of BRG1, and not by the more modest decrease in nuclear GR levels.

We sought to correlate gene expression changes in A1A3 cells with the presence and proximity of GR peaks. DEG TSSs tended to be closer to GR peaks, and the percentage of DEGs with GR peaks within 50 kb was more than double that of non-DEG obsTSSs (77.2 to 29.8%). Thus, while GR binding is distal from gene TSSs, genes that are regulated by GR tend to have a higher degree of local GR binding sites. When considering the closest GR peak to each DEG, the different types of DEGs had different proportions of GR peaks classes, with Class II peaks enriched among the closest peaks to ‘common’ and ‘gained’ DEGs (Figure 3D). Comparing the distance from the closest GR peak to each DEG TSS, Class II peaks were also closer than Class I or Class III peaks (Figure 3E). Overall, ‘common’ DEGs had closer GR peaks than ‘gained’ or ‘lost’ DEGs, with the median distance from TSSs to closest GR peaks of 6889, 20094, and 22104 bp, respectively (Figure 3F). DEGs that were ‘gained’ or ‘lost’ also had fewer GR peaks within 50 kb of their TSSs (Figure 3G). Taken together, these data indicate that BRG1 presence was more critical for hormone responsiveness at genes where GR binding was the most distal.

### GR peak classes have distinct underlying DNA sequences and transcription factor motifs

**Figure 4.**
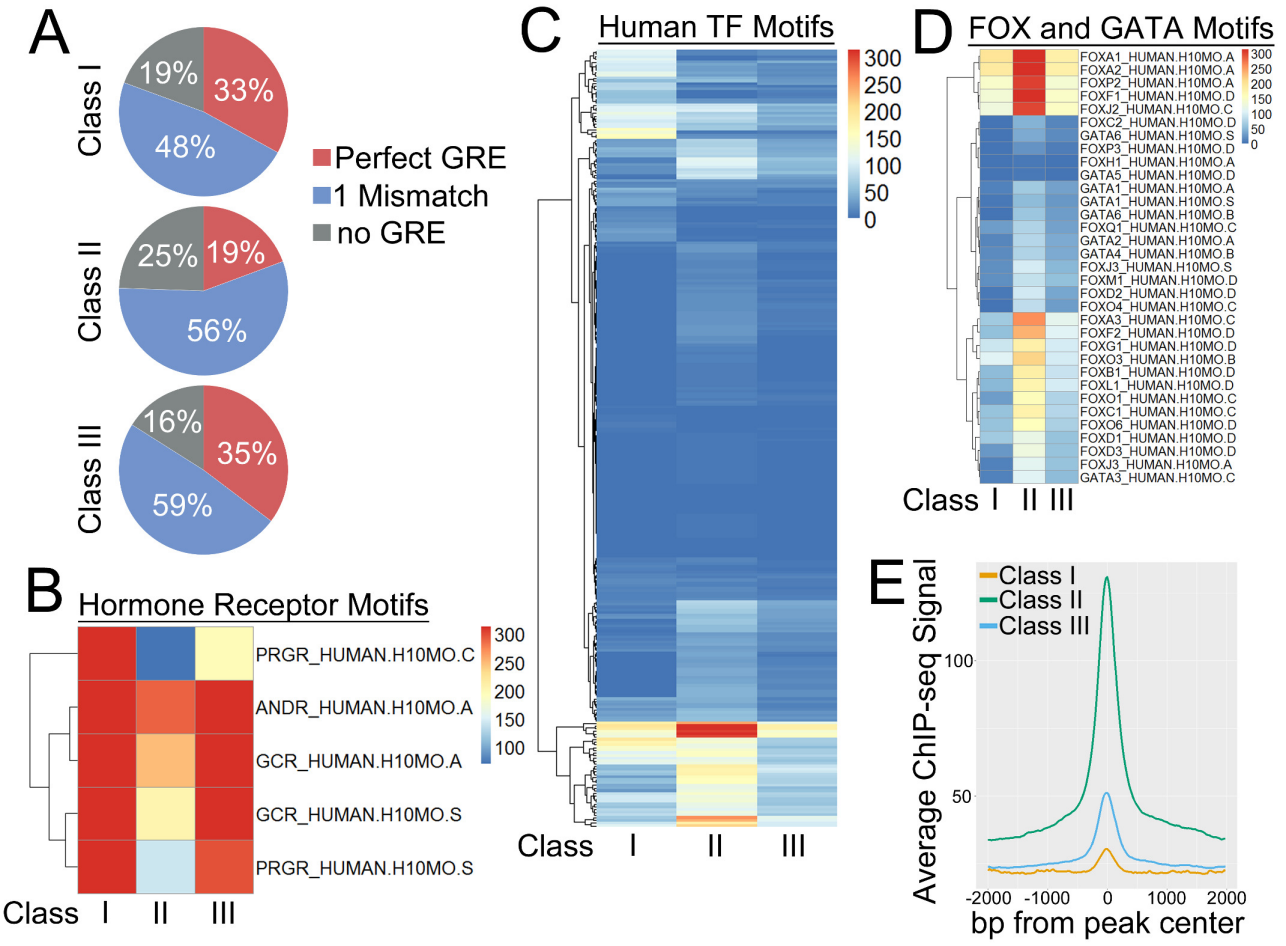
GR peak classes have distinct underlying DNA sequences and transcription factor motifs. (**A**) Class II GR peaks have fewer perfect GRE motifs than Class I or Class III peaks. (**B**) Heatmap of -log10 p-values for enrichment of hormone receptor motifs under GR peak classes. (**C**) Heatmap of -log10 p-values for enrichment of human transcription factor motifs under GR peak classes. (**D**) Heatmap of -log10 p-values for enrichment of FOX and GATA motifs under GR peak classes. (**E**) Meta-profile of average coverage of 25 ENCODE transcription factor ChIP-seq MCF7 datasets over GR peak classes. 10.7554/eLife.35073.014Figure 4—source data 1.Table of MCF7 ENCODE ChIP-seq data sets.Table of ENCODE ChIP-seq data sets from MCF7 cells used to calculated average transcription factor ChIP-seq meta-plot in Figure 4E.

GR frequently binds to degenerate GR recognition sequences or GR response elements (GREs) (Starick et al., 2015) and can also interact with other regions of the genome through cooperation with or tethering by other transcription factors such as AP-1, NFκB, and STAT proteins (Biddie et al., 2011; Rao et al., 2011; Langlais et al., 2012). To determine whether our GR peaks classes segregated distinct sequence specificities, we first searched under GR peaks for perfect GREs. Using the total set of GR peaks, perfect GREs were found under 28.3% of the peaks and GREs with single mismatches were found under an additional 51% of peaks (Figure 4A). Perfect GREs were more common in Class I and Class III peaks (33 and 35.3%, respectively) than Class II peaks (19.4%) (Figure 4A). Motif analysis revealed similar patterns, with GREs, Androgen Receptor motifs, and Progesterone Receptor motifs being strongly enriched under all three peak classes, but with the lowest enrichment levels under Class II peaks (Figure 4B).

**Figure 4—figure supplement 1.**
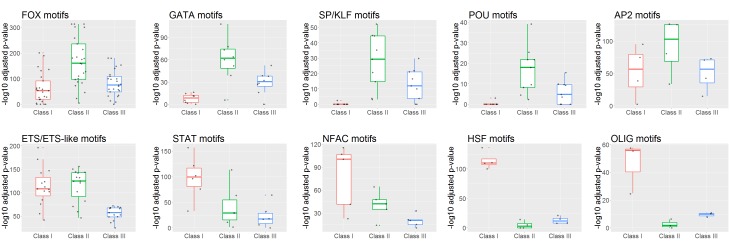
Transcription factor motifs enriched under distinct GR peak classes. Box plots of -log10 p-values of families of transcription factor motifs under Class I, II, and III GR peaks. Note that Fox, Gata, SP/KLF, and Pou family motifs are most strongly enriched under Class II peaks, while Stat, NFAC, Olig, and HSF motifs are most strongly enriched under Class I peaks.

Conversely, Class II peaks were most strongly enriched for other transcription factors. Motif analysis revealed that Class II peaks exhibited the highest average level of motif enrichment (Figure 4C). This was largely driven by FOX and GATA motifs, which were more strongly enriched under Class II peaks than Class I or Class III peaks (Figure 4D). To validate these predictive analyses, we pulled data from 25 ENCODE transcription factor ChIP-seq experiments in Mcf7 cells (Figure 4—source data 1) and generated a meta-profile of transcription factor ChIP enrichment over the three GR peak classes (Figure 4E). Class II GR peaks displayed the strongest levels of enrichment, while Class I and Class III peaks showed only low to moderate levels of enrichment (Figure 4E). Motif analyses yielded several other interesting motif families to consider in the context of BRG1 and the GR peak classes. SP/KLF and POU motifs were specifically enriched under Class II and Class III peaks (Figure 4—figure supplement 1), which suggested that GR binding in cooperation with these transcription factor families may also involve BRG1. On the other hand, STAT, NFATC, and OLIG motifs were most strongly enriched under Class I GR peaks (Figure 4—figure supplement 1), indicative of transcription factor interactions that may occur in the absence of BRG1. Taken together, these analyses revealed that the three GR peak classes had distinguishable DNA sequence content and that the BRG1-GR interaction could be moderated by other transcription factors.

### BRG1 is required for Dex-induced recruitment of pioneer factors to GR binding sites

**Figure 5.**
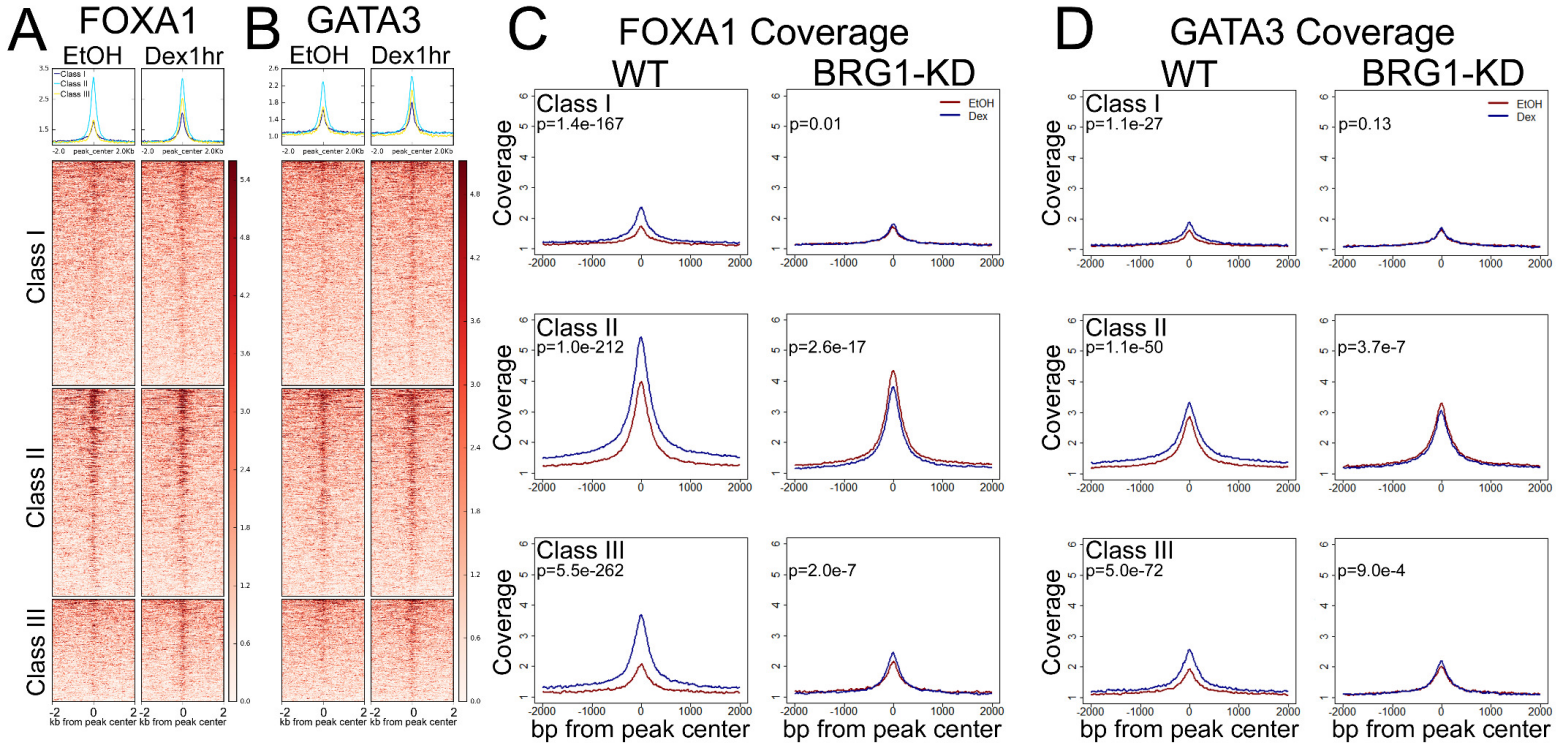
BRG1 is required for Dex-induced recruitment of pioneer factors. (**A–B**) FOXA1 and GATA3 ChIP-seq coverage is enriched across all GR peak classes, but shows strongest enrichment over Class II peaks. (**C–D**) Meta-profiles of FOXA1 and GATA3 ChIP-seq signal over GR peak classes in A1A3 cells. In wild-type cells, Dex treatment induced recruitment of additional FOXA1 and GATA3 to all three GR peak classes. This recruitment is lost following BRG1-KD. To calculate P-values in (**C**) and (**D**), we used the average signal in the 500 bp window centered on the center of the GR peak, and performed unpaired Wilcoxon Rank Sum/Mann-Whitney test.

Recent work has suggested that the hormone response is coordinated by functional interactions between nuclear hormone receptors and pioneer factors such as FOXA1 and GATA3 (Biddie et al., 2011; Grøntved et al., 2013; Carroll et al., 2005; Holmqvist et al., 2005; Laganiere et al., 2005; Hurtado et al., 2011). As we observed differential enrichment of FOXA1 and GATA3 motifs under the GR peak classes, we performed ChIP-seq to examine the interaction of these factors at each peak class. Both FOXA1 and GATA3 showed strong levels of enrichment at Class II GR peaks in both untreated and 1 hr Dex treated cells (Figure 5A–B). Class I and Class III peaks had similarly low levels of FOXA1 and GATA3 in untreated cells (Figure 5A–B). However, at Class III peaks, there was a marked increase in the detected levels of FOXA1 and GATA3 binding upon 1 hr of Dex treatment (Figure 5A–B) comparable to the Dex-induced enrichment of BRG1 at these peaks (Figure 1E). Thus, at GR peaks, the pattern of pioneer factor binding correlated with that of BRG1 binding.

To determine whether BRG1 was required for pioneer factor binding at GR peaks, we performed GR and FOXA1 ChIP-seq in A1A3 cells. The levels of FOXA1 and GATA3 protein were similar between control and BRG1-KD cells (Figure 3—figure supplement 1), indicating that BRG1 was not required for their expression. In control cells treated with Dex for 1 hr, a Dex-induced increase in FOXA1 and GATA3 enrichment was observed at all three GR peak classes, with the most substantial increase occurring at Class III peaks (Figure 5C–D, left columns)). In vehicle treated BRG1-KD cells, the loss of BRG1 appeared to have little effect on the enrichment of FOXA1 and GATA3 at GR peaks (Figure 5C–D, red lines). In contrast, the DEX-induced increase in FOXA1 and GATA3 enrichment at GR peaks was almost completely blocked in BRG1-KD cells (Figure 5C–D, blue lines). Taken together, these experiments demonstrate that BRG1 was not required for pioneer factor interaction at GR peaks. However, BRG1 was required for hormone-induced changes in pioneer factor enrichment at GR peaks.

### BRG1 binding to pioneer factor peaks is predictive of GR binding upon hormone treatment

**Figure 6.**
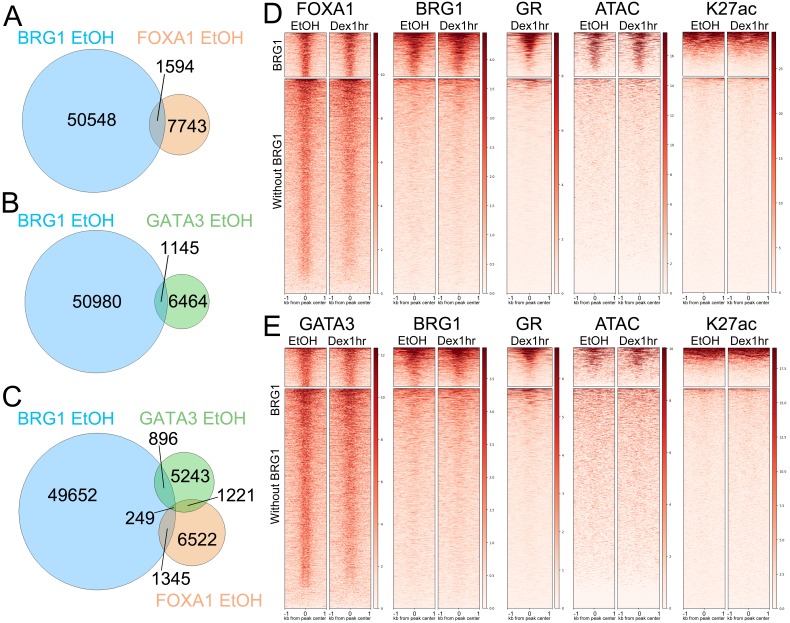
BRG1 binding to pioneer factor binding sites is predictive of GR binding upon Dex treatment. (**A**) Overlap between BRG1 EtOH and FOXA1 EtOH peaks. (**B**) Overlap between BRG1 EtOH and GATA3 EtOH peaks. (**C**) Three-way Venn diagram showing overlap of all three factors (**D**) Heatmaps depicting FOXA1, BRG1, GR, ATAC nucleosome free reads, and K27ac over FOXA1 EtOH peaks divided into ‘with BRG1’ and ‘without BRG1’ subsets. (**E**) Heatmaps depicting GATA3, BRG1, GR, ATAC nucleosome free reads, and K27ac over GATA3 EtOH peaks divided into ‘with BRG1’ and ‘without BRG1’ subsets. Note that in both (**D**) and (**E**) GR binding to FOXA1 and GATA3 peaks is largely restricted to ‘with BRG1’ peaks.

We next sought to take a pioneer factor-centric approach to determine whether the presence of BRG1 affected the interaction of GR with pioneer factors. In vehicle-treated cells, BRG1 peaks intersected 16.4% of FOXA1 peaks (1594 peaks, Figure 6A,D) and 14.5% of GATA3 peaks (1145 peaks, Figure 6B,E). These peaks were predominantly unique, with only 249 of the FOXA1 +BRG1 peaks intersecting a GATA3 +BRG1 peak, similar to the overall proportion of overlap between FOXA1 and GATA3 peaks (Figure 6C). For both FOXA1 and GATA3, perfect GRE motifs were present at similar proportions between peaks with or without BRG1, with approximately 31% of FOXA1 peaks and 28% of GATA3 peaks having a perfect GRE motif within 500 bp of the center of the peak (Figure 6—figure supplement 1). Despite this, GR binding at FOXA1 and GATA3 peaks was almost entirely restricted to peaks that intersected BRG1 peaks (Figure 6D–E). As BRG1 was present at these sites in both untreated and Dex-treated cells (Figure 6D–E), these are Class II GR peaks. ATAC-seq nucleosome-free reads and K27ac ChIP-seq signal were also predominantly restricted to pioneer factor peaks that intersected BRG1 (Figure 6D–E). Thus, the presence of BRG1 at pioneer factor peaks in untreated cells was predictive of subsequent GR binding upon Dex treatment. These findings suggest that BRG1 is involved in pre-patterning a subset of pioneer factor binding sites to facilitate GR binding upon hormone treatment and that pioneer factor binding alone is not predictive of GR binding. Class II GR peaks represent GR binding to regions of chromatin that are pre-patterned by BRG1 and pioneer factors.

**Figure 6—figure supplement 1.**
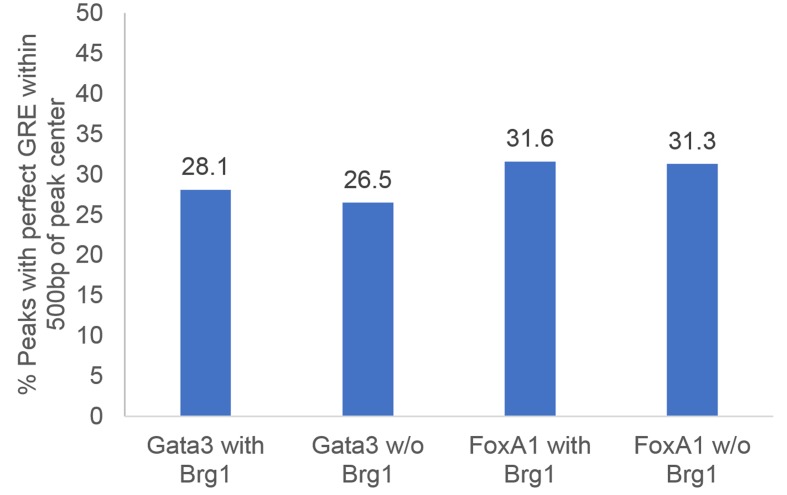
Proportion of GREs under pioneer factor peaks. Percentage of GATA3 and FOXA1 peaks that have perfect GRE motifs within 500 bp of the center of the peak. The percentage of peaks with perfect GRE motifs is similar between peaks ‘with BRG1’ and peaks ‘without BRG1.’.

## Discussion

**Figure 7.**
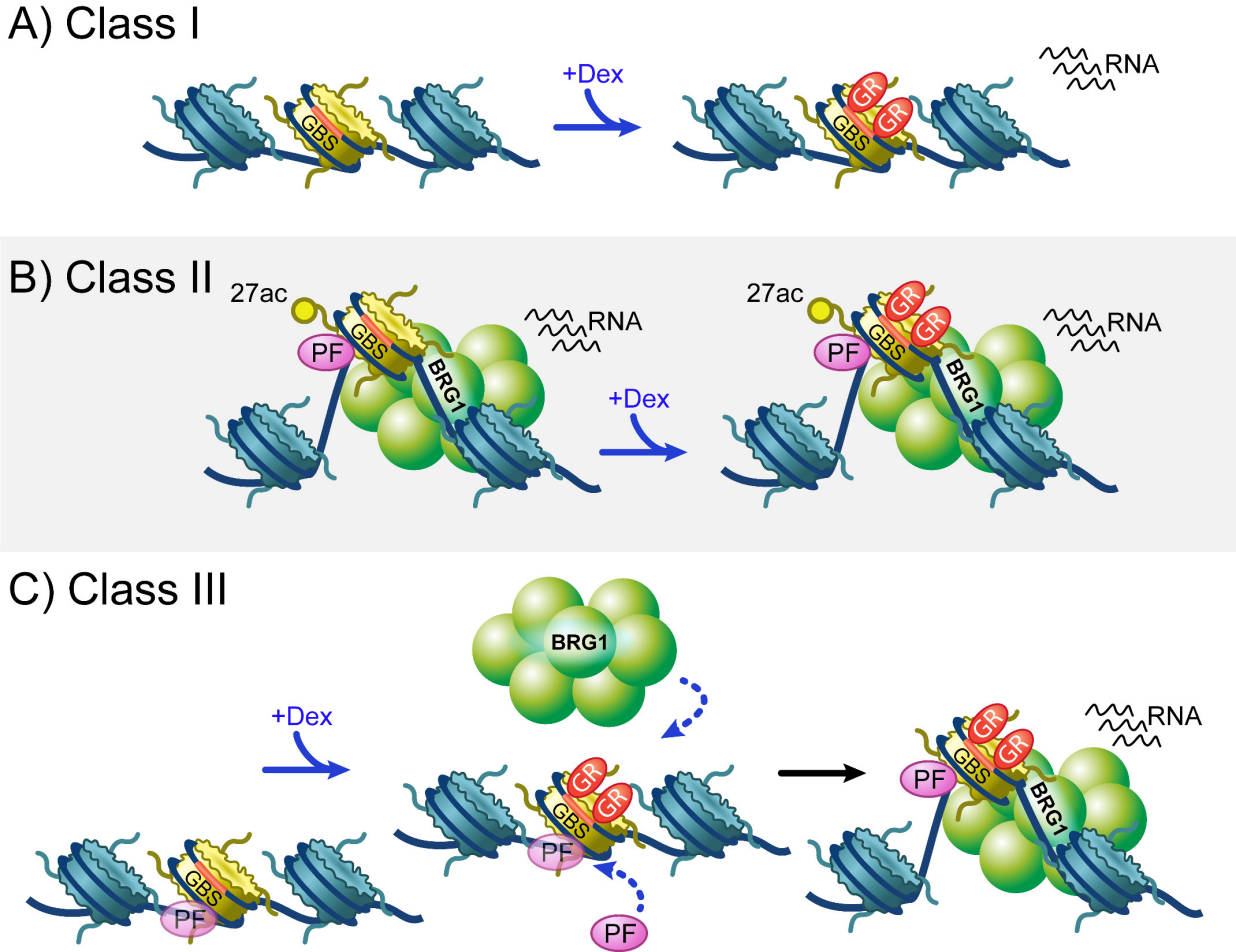
Overview of three classes of GR binding site. (**A**) Class I GR binding sites (GBSs) reside within relatively closed regions of chromatin and have little dex-dependent chromatin remodeling or recruitment of BRG1 and pioneer factors. Despite this, greater than a quarter of Class I GBSs exhibit dex-dependent transcription. (**B**) Class II GBSs represent GR binding to active and occupied regions of chromatin. These GBSs are bound by BRG1 prior to hormone treatment, and also exhibit hormone independent H3K27 acetylation, chromatin accessibility, pioneer factor binding, and transcriptional activity. (**C**) Class III GBSs behave the most like the model described at the MMTV promoter. Upon hormone treatment, GR binds to regions of relatively inaccessible chromatin that may be pre-occupied by low levels of pioneer factors. Upon GR binding, BRG1 and additional pioneer factors are recruited, chromatin remodeling yields increased accessibility, and more than a third of these GBSs gain transcriptional activity.

The requirement for BRG1-mediated chromatin remodeling in potentiating the transcriptional response to glucocorticoid signaling at model genes (e.g. MMTV) was established over two decades ago. Our examination of the genomic glucocorticoid response demonstrates a previously undescribed role of BRG1 in patterning the underlying chromatin architecture. Our data reveals that BRG1 interacts with approximately 40% of GR binding sites prior to hormone treatment, and an additional 20% of GR binding sites upon hormone treatment. BRG1 is also broadly associated with transcriptional activity at active gene and enhancer TSSs. The patterns of BRG1 binding at GR biding sites prior to and upon hormone signaling allowed us to define three classes of GR binding site (Figure 7). These classes exhibited distinct patterns of underlying chromatin accessibility, transcriptional activity, histone modification, and transcription factor motif enrichment and binding. These findings are corroborated by the observation that GR bound enhancers exist in three distinct chromatin states in mouse mammary adenocarcinoma cells (Johnson et al., 2018). Class I and Class III peaks gain chromatin accessibility upon Dex exposure and are associated with Dex-specific enhancer TSSs, suggesting that they represent regulatory elements that are activated only upon hormone treatment. Class II GR binding sites are strikingly enriched for chromatin that is active and accessible prior to hormone signaling and appear to represent GR binding to active enhancers.

Our examination of GR binding sites yielded several interesting observations regarding the nature of GR binding and transcriptional activity. The majority of GR binding sites are not associated with transcriptional activity, indicating that GR binding to chromatin is not sufficient to activate transcription. This is especially evident at Class I peaks, which are devoid of active chromatin markers and display minimal ATAC accessibility. Active and accessible chromatin was only detected at Class II GR peaks where BRG1 localization was constitutive/hormone-independent. This active chromatin environment was pre-existing, and was largely unchanged by Dex exposure and GR binding. However, half of Class II peaks do not have active TSSs within 1 kb, and not all Class II peaks have appreciable K27ac enrichment. Furthermore, BRG1 binding was also not sufficient to generate a fully activated chromatin environment at GR binding sites, as Dex-induced recruitment of BRG1 at Class III peaks occurs without a concomitant induction of K27ac. The implications for these phenomena are wide-ranging. Enrichment of H3K4me1 at Class I and III GR peaks suggests that these binding sites are poised for transcriptional activation, and yet, Dex-induced binding of GR and BRG1 do not yield conversion of these sites to a more active chromatin profile such as that observed at Class II peaks. Thus, it appears that a large subset of GR chromatin interactions are transcriptionally unproductive and uneventful in terms of the effect on the chromatin environment. Despite this, Start-seq reveals that transcriptional activity is gained at ~25% of Class I peaks and ~30% of Class III peaks upon Dex exposure. This suggests that the induction of transcriptional activity at GR binding sites occurs independently of the induction of common active chromatin characteristics.

Intriguingly, ~60% of Dex-regulated DEGs are lost following BRG1 knockdown, and Class II and III GR binding sites represent ~60% of GR binding sites. The suppression of the GR transcriptional response following BRG1 knockdown suggested that BRG1 interaction with GR binding sites is required for GR-mediated transcriptional regulation of many genes. Surprisingly, over 100 genes gained hormone-responsiveness following BRG1 knockdown, indicating that at some genes, BRG1 prevents GR from eliciting transcriptional activity. Thus, BRG1 plays a critical role in patterning the GR transcriptional response. As the number of GR peaks is substantially greater than the number of GR-regulated genes, and most genes have multiple GR peaks of different classes within the surrounding several hundred kilobases, it is difficult to clearly associate individual GR peaks or peak classes with specific genes. The distal nature of GR binding events and the enhancer-like characteristics of the chromatin under GR binding sites indicate that the GR signaling largely regulates transcription through modulation of enhancer activity. On the other hand, BRG1 was enriched at most active gene and enhancer TSSs, implicating a widespread role for BRG1 in facilitating transcriptional activity. A reasonable hypothesis for GR signaling would be that BRG1 binding at gene TSSs and GR binding sites/enhancers promotes chromatin looping. Long-range chromatin interactions have been implicated in GR transcriptional activity (Vockley et al., 2016; Hakim et al., 2009). It has also been suggested that clusters of GBSs interact with each other over long ranges to synergistically regulate transcription of target genes Holmqvist et al., 2005; Vockley et al., 2016). Identifying such long-range interactions between GR and BRG1 would provide more insight into whether the different classes of GR binding sites are differentially utilized in regulating transcription. Such long range interactions could potentially provide functional rationale for the existence of ‘unproductive’ GR binding sites, which could be associated with transcriptionally active GR binding sites to cooperatively regulate transcriptional output. Alternatively, GR and BRG1 could regulate gene expression through decompaction of broad regions of chromatin surrounding gene TSSs and enhancers, such as has been reported at the Fkbp5 and Ms4xxx loci in macrophages (Jubb et al., 2017). In either case, removing individual or combinations of GR peaks around candidate DEGs will allow for interrogation of how multiple GR binding events are coordinated to elicit a transcriptional response. The eventual identification of which GR binding sites are critical for transcriptional regulation in a given cell type will have wide-ranging implications for the pharmacological targeting of GR signaling for disease treatment.

We observed that BRG1 binding at Class II binding sites is predictive of potential GR interactions with pioneer factor binding sites. This finding is intriguing when considered along with the recent finding that FOXA1 binding is reorganized upon activation of ER or GR (Swinstead et al., 2016). Like FOXA1, BRG1 binding is significantly reorganized upon hormone treatment and the majority of EtOH-specific and Dex-specific BRG1 peaks are not associated with GR binding sites [Figure 1, Figure 1—figure supplement 1]. Thus, the rearrangement of BRG1 and FOXA1 binding does not appear to be dependent on direct interaction with GR. While these rearrangements occur within an hour of hormone treatment, it is possible that the reorganization of BRG1 and FOXA1 binding occurs secondarily to the initial GR binding events, which occur rapidly within the first several minutes of hormone treatment (unpublished data). Alternatively, it is also possible that a subset of interactions between GR, BRG1 and FOXA1 on chromatin are not detectable by standard ChIP methods. Single molecule analysis of GR, BRG1, and FOXA1 indicated that the majority of chromatin interactions occur with residence times of approximately 1 s, and a minority of events occur with longer residence times of 5 to 10 s (Swinstead et al., 2016; Paakinaho et al., 2017). Thus, chromatin binding by these factors in individual cells is highly dynamic. While ChIP-seq experiments represent the overall binding profile of these factors in large populations of cells, they still represent snapshots of the chromatin interaction profiles of these factors and could fail to detect the full expanse of rapid and dynamic binding events. As such, it remains unclear whether there is any set hierarchy of binding or timing/order of events of FOXA1, BRG1, and GR chromatin interactions. As suggested by the distinct patterns of interactions at GR peak classes, it is likely that several series of binding events occur at GR binding sites prior to and upon hormone signaling. Elucidating these distinct mechanisms will help to unravel the basic mechanisms of nuclear receptor signaling and the role of pioneer factors and chromatin remodeling complexes in facilitating chromatin interactions and modulating transcriptional output.

## Materials and methods

**Key resources table:** 

Reagent type (species) or resource	Designation	Source or reference	Identifiers	Additional information
Cell line (*H.sapiens*)	A1-2	PMID: 7838148	RRID:CVCL_0I95	T47D derivative with incorporated rat GR
Cell line (*H.sapiens*)	A1-A3	PMID: 22451486		A1-2 derivative with incorporated BRG1 shRNA
Antibody	anti-BRG1	PMID: 26055322		lab-made, ChIP = 1 ug/100 ug chromatin, Western Blot = 0.1 ug/ml
Antibody	anti-GR	Santa Cruz	M-20, sc-1004, RRID:AB_2155786	ChIP = 1 ug/100 ug chromatin, Western Blot = 0.1 ug/ml
Antibody	anti-FOXA1	Abcam	ab23738, RRID:AB_2104842	ChIP = 1 ug/100 ug chromatin, Western Blot = 0.1 ug/ml
Antibody	anti-GATA3	Cell Signaling	D13C9, RRID:AB_10834528	ChIP = 1 ug/100 ug chromatin, Western Blot = 0.1 ug/ml
Antibody	anti-H3K27ac	Abcam	ab4729, RRID:AB_2118291	ChIP = 1 ug/100 ug chromatin
Antibody	anti-H3K27me3	Active Motif	39155, RRID:AB_2561020	ChIP = 1 ug/100 ug chromatin
Antibody	anti-H3K4me1	Abcam	ab8895, RRID:AB_306847	ChIP = 1 ug/100 ug chromatin
Antibody	anti-KU70	Santa Cruz	H-308, sc-9033, RRID:AB_650476	Western Blot = 0.1 ug/ml
Sequence-based reagent	GR siRNA	Dharmacon	ON-TARGETplus J-089504–07	UUACAAAGAUUGCAGGUAU
Sequence-based reagent	Non-targeting Control siRNA	Dharmacon	ON-TARGETplus Not-targeting Pool D-001810-10-20	
Commercial assay or kit	Nextera XT library generation kit	Illumina	15032350	
Commercial assay or kit	SuperScript III First-Strand kit	Invitrogen	18080–051	
Commercial assay or kit	iScript cDNA Sythesis kit	Bio-Rad	170–8891	
Commercial assay or kit	ssoAdvanced Universal SYBR Green Supermix	Bio-Rad	172–5274	
Commercial assay or kit	RNeasy Mini Kit	Qiagen	74104	
Commercial assay or kit	RNA 6000 RNA Pico Kit	Agilent Technologies	5067–1513	
Commercial assay or kit	QiaQuick PCR purification kit	Qiagen	28104	
Commercial assay or kit	HALT protease inhibitors	ThermoFisher	78430	
Chemical compound, drug	Dexamethasone	Sigma	D4902	100 nM
Chemical compound, drug	Doxycycline	Sigma	D9891	10 ug/ml
Software, algorithm	Cutadapt	DOI: http://dx.doi.org/10.14806/ej.17.1.200	RRID:SCR_011841	
Software, algorithm	Sickle	https://github.com/najoshi/sickle	RRID:SCR_006800	
Software, algorithm	Bowtie2	PMID: 22388286	RRID:SCR_005476	
Software, algorithm	Samtools	PMID: 19505943	RRID:SCR_002105	
Software, algorithm	MACS2	PMID: 18798982	RRID:SCR_013291	
Software, algorithm	Homer	PMID: 20513432	RRID:SCR_010881	
Software, algorithm	Bedtools	PMID: 20110278	RRID:SCR_006646	
Software, algorithm	Deeptools	PMID: 27079975		
Software, algorithm	AME	DOI: https://doi.org/10.1186/1471-2105-11-165	RRID:SCR_001783	http://meme-suite.org/tools/ame
Software, algorithm	STAR	PMID: 23104886	RRID:SCR_015899	
Software, algorithm	Salmon	PMID: 28263959		
Software, algorithm	limma-voom	PMID: 24485249	RRID:SCR_010943	

### Cell culture

T47D derived A1-2 (Archer et al., 1994) and A1-A3 (Burd et al., 2012) cells were cultured as previously described (Burd et al., 2012). Both cell lines were authenticated by STR profiling and tested negative for mycoplasma. Dexamethasone treatments were performed using 100 nM Dexamethasone or ethanol vehicle for 1 or 8 hr for ChIP-seq and RNA-seq experiments, respectively. To knockdown BRG1 expression in A1-A3 cells, cells were treated for 72 hr with Doxycycline.

### ChIP and ChIP-seq

ChIP experiments were largely performed as previously described (Takaku et al., 2016). Cells were fixed with 1% formaldehyde at 37C for 10 min for all targets except BRG1, for which cells were fixed for 20 min. After quenching with glycine, cell pellets were washed Hypotonic buffer (10 mM HEPES-NaOH pH 7.9, 10 mM KCl, 1.5 mM MgCl2, 340 mM sucrose, 10% glycerol, 0.1% Triton X-100, and HALT protease inhibitors (ThermoFisher)) and resuspended in Shearing buffer (10 mM Tris-HCl pH 8.0, 1 mM EDTA, 0.5 mM EGTA, 0.5 mM PMSF, 5 mM Sodium Butyrate, 0.1% SDS, and HALT protease inhibitors (ThermoFisher) and chromatin was fragmented by sonication with the Covaris S220. Chromatin was diluted two-fold in 2xIP buffer (20 mM Tris-HCl pH 8.0, 300 mM NaCl, 2 mM EDTA, 20% Glycerol, 1% Triton X-100, 0.5 mM PMSF, 5 mM Sodium Butyrate, and HALT protease inhibitors (ThermoFisher)) and immunoprecipitation was performed with antibodies specific to BRG1 (lab-made, [Trotter et al., 2015]), GR (Santa Cruz M-20), FOXA1 (Abcam ab23738), GATA3 (Cell Signaling D13C9), and H3K27ac (Abcam ab4729) and ratios of 1 ug antibody per 100 ug chromatin. Immune complexes were captured using protein A and G dynabeads, washed once each with low salt (20 mM Tris-HCl pH 8.0, 150 mM NaCl, 2 mM EDTA, 1% Triton X-100, 0.1% SDS), high salt (same as low salt buffer, except 500 mM NaCl), and LiCl buffer (Tris-HCl pH 8.0, 250 mM LiCl, 2 mM EDTA, 1 % NP-40, 1% (wt/vol) sodium deoxycholate), and twice with TE. Eluted DNA was RNaseA and Proteinase K treated and purified using Qiagen PCR purification columns. ChIP-seq libraries were generated using the Illumina Nextara-XT library generation kit, and sequenced on the Illumina MiSeq and NextSeq platforms. For all ChIP-seq experiments, biological duplicates or triplicates were performed, and all presented ChIP-seq data are representative single experimental replicates. Examples of reproducibility of multiple replicates are presented in Figure 1—figure supplement 2.

Adapter sequences were trimmed from ChIP-seq reads using Cutadapt (Martin, 2011) and low quality reads were removed from analysis using Sickle (Joshi NA et al., 2011). Alignment was performed with Bowtie2 (Langmead and Salzberg, 2012). Aligned reads were sorted and processed with Samtools (Li et al., 2009) and de-duplicated using Picard Tools (http://broadinstitute.github.io/picard). Peaks were called using MACS2 (Zhang et al., 2008) and Homer (Heinz et al., 2010) using a false discovery rate cutoff of 0.001, and regions of high depth or with high signal in untreated or input samples were used to filter out false positive peak calls. Peak overlaps and distance analyses were performed using Bedtools (Quinlan and Hall, 2010). Coverage files and heatmaps were generated using Deeptools (Ramírez et al., 2016). Motif analyses were performed using AME (McLeay and Bailey, 2010).

### RNA isolation, cDNA synthesis, QPCR, and RNA-seq

RNA was isolated from treated A1-2 and A1A3 cells using Qiagen RNeasy kits with on-column DNase treatment. ThermoFisher SuperScript III or BioRad iScript were used to synthesize DNA and qPCR was run with BioRad ssoAdvanced Universal SYBR Green Supermix. For RNA-seq, RNA quality was validated with RNA 6000 RNA Pico Kit on the Agilent Bioanalyzer 2100. RNA-seq libraries were generated at the National Intramural Sequencing Center using Ribo-Zero Gold and sequenced on an Illumina HiSeq 2500. Adapter sequences were trimmed from RNA-seq reads using Cutadapt (Martin, 2011) and low quality reads were removed from analysis using Sickle (Joshi NA et al., 2011). Alignment was performed using STAR (Dobin et al., 2013) to generate coverage tracks and using Salmon (Patro et al., 2017) and to obtain gene counts for differential expression analysis using limma-voom (Law et al., 2014) with cutoffs of Fold Change > 1.5, p-value<0.01, and False Discovery Rate < 0.05.

### Data availability

All ChIP-seq and RNA-seq data generated for this publication have been deposited in NCBI's Gene Expression Omnibus (Edgar et al., 2002) and are accessible through GEO Series accession number GSE112491 (https://www.ncbi.nlm.nih.gov/geo/query/acc.cgi?acc=GSE112491).

## Additional files

Transparent reporting form

### Data availability

Sequencing data have been deposited in GEO under accession code GSE112491.

The following dataset was generated:

Jackson A HoffmanKevin W TrotterJames M WardTrevor K Archer2018BRG1 governs Glucocorticoid Receptor interactions with chromatin and pioneer factors across the genomehttps://www.ncbi.nlm.nih.gov/geo/query/acc.cgi?acc=GSE112491Publicly available at the NCBI Gene Expression Omnibus (accession no: GSE112491)

## References

[bib1] Archer TK, Zaniewski E, Moyer ML, Nordeen SK (1994). The differential capacity of glucocorticoids and progestins to alter chromatin structure and induce gene expression in human breast cancer cells. Molecular Endocrinology.

[bib2] Biddie SC, John S, Sabo PJ, Thurman RE, Johnson TA, Schiltz RL, Miranda TB, Sung MH, Trump S, Lightman SL, Vinson C, Stamatoyannopoulos JA, Hager GL (2011). Transcription factor AP1 potentiates chromatin accessibility and glucocorticoid receptor binding. Molecular Cell.

[bib3] Burd CJ, Ward JM, Crusselle-Davis VJ, Kissling GE, Phadke D, Shah RR, Archer TK (2012). Analysis of chromatin dynamics during glucocorticoid receptor activation. Molecular and Cellular Biology.

[bib4] Carroll JS, Liu XS, Brodsky AS, Li W, Meyer CA, Szary AJ, Eeckhoute J, Shao W, Hestermann EV, Geistlinger TR, Fox EA, Silver PA, Brown M (2005). Chromosome-wide mapping of estrogen receptor binding reveals long-range regulation requiring the forkhead protein FoxA1. Cell.

[bib5] Cordingley MG, Riegel AT, Hager GL (1987). Steroid-dependent interaction of transcription factors with the inducible promoter of mouse mammary tumor virus in vivo. Cell.

[bib6] Dobin A, Davis CA, Schlesinger F, Drenkow J, Zaleski C, Jha S, Batut P, Chaisson M, Gingeras TR (2013). STAR: ultrafast universal RNA-seq aligner. Bioinformatics.

[bib7] Edgar R, Domrachev M, Lash AE (2002). Gene expression omnibus: ncbi gene expression and hybridization array data repository. Nucleic Acids Research.

[bib8] Fryer CJ, Archer TK (1998). Chromatin remodelling by the glucocorticoid receptor requires the BRG1 complex. Nature.

[bib9] Grøntved L, John S, Baek S, Liu Y, Buckley JR, Vinson C, Aguilera G, Hager GL (2013). C/EBP maintains chromatin accessibility in liver and facilitates glucocorticoid receptor recruitment to steroid response elements. The EMBO Journal.

[bib10] Hakim O, John S, Ling JQ, Biddie SC, Hoffman AR, Hager GL (2009). Glucocorticoid receptor activation of the *Ciz1-Lcn2* locus by long range interactions. Journal of Biological Chemistry.

[bib11] Heinz S, Benner C, Spann N, Bertolino E, Lin YC, Laslo P, Cheng JX, Murre C, Singh H, Glass CK (2010). Simple combinations of lineage-determining transcription factors prime cis-regulatory elements required for macrophage and B cell identities. Molecular Cell.

[bib12] Holmqvist PH, Belikov S, Zaret KS, Wrange O (2005). FoxA1 binding to the MMTV LTR modulates chromatin structure and transcription. Experimental Cell Research.

[bib13] Hsiao PW, Fryer CJ, Trotter KW, Wang W, Archer TK (2003). BAF60a mediates critical interactions between nuclear receptors and the BRG1 chromatin-remodeling complex for transactivation. Molecular and Cellular Biology.

[bib14] Hurtado A, Holmes KA, Ross-Innes CS, Schmidt D, Carroll JS (2011). FOXA1 is a key determinant of estrogen receptor function and endocrine response. Nature Genetics.

[bib15] Inoue H, Furukawa T, Giannakopoulos S, Zhou S, King DS, Tanese N (2002). Largest subunits of the human SWI/SNF chromatin-remodeling complex promote transcriptional activation by steroid hormone receptors. Journal of Biological Chemistry.

[bib16] John S, Sabo PJ, Thurman RE, Sung MH, Biddie SC, Johnson TA, Hager GL, Stamatoyannopoulos JA (2011). Chromatin accessibility pre-determines glucocorticoid receptor binding patterns. Nature Genetics.

[bib17] Johnson TA, Chereji RV, Stavreva DA, Morris SA, Hager GL, Clark DJ (2018). Conventional and pioneer modes of glucocorticoid receptor interaction with enhancer chromatin in vivo. Nucleic Acids Research.

[bib18] Johnson TA, Elbi C, Parekh BS, Hager GL, John S (2008). Chromatin remodeling complexes interact dynamically with a glucocorticoid receptor-regulated promoter. Molecular Biology of the Cell.

[bib19] Joshi NA, Fass JN (2011). https://github.com/najoshi/sickle.

[bib20] Jubb AW, Boyle S, Hume DA, Bickmore WA (2017). Glucocorticoid receptor binding induces rapid and prolonged large-scale chromatin decompaction at multiple target loci. Cell Reports.

[bib21] Kadoch C, Hargreaves DC, Hodges C, Elias L, Ho L, Ranish J, Crabtree GR (2013). Proteomic and bioinformatic analysis of mammalian SWI/SNF complexes identifies extensive roles in human malignancy. Nature Genetics.

[bib22] Laganiere J, Deblois G, Lefebvre C, Bataille AR, Robert F, Giguere V (2005). Location analysis of estrogen receptor target promoters reveals that FOXA1 defines a domain of the estrogen response. PNAS.

[bib23] Langlais D, Couture C, Balsalobre A, Drouin J (2012). The Stat3/GR interaction code: predictive value of direct/indirect DNA recruitment for transcription outcome. Molecular Cell.

[bib24] Langmead B, Salzberg SL (2012). Fast gapped-read alignment with Bowtie 2. Nature Methods.

[bib25] Lavender CA, Cannady KR, Hoffman JA, Trotter KW, Gilchrist DA, Bennett BD, Burkholder AB, Burd CJ, Fargo DC, Archer TK (2016). Downstream antisense transcription predicts genomic features that define the specific chromatin environment at mammalian promoters. PLOS Genetics.

[bib26] Law CW, Chen Y, Shi W, Smyth GK (2014). voom: precision weights unlock linear model analysis tools for RNA-seq read counts. Genome Biology.

[bib27] Li H, Handsaker B, Wysoker A, Fennell T, Ruan J, Homer N, Marth G, Abecasis G, Durbin R, 1000 Genome Project Data Processing Subgroup (2009). The sequence alignment/map format and SAMtools. Bioinformatics.

[bib28] Martin M (2011). Cutadapt removes adapter sequences from high-throughput sequencing reads. EMBnet.journal.

[bib29] McLeay RC, Bailey TL (2010). Motif Enrichment Analysis: a unified framework and an evaluation on ChIP data. BMC Bioinformatics.

[bib30] Nie Z, Xue Y, Yang D, Zhou S, Deroo BJ, Archer TK, Wang W (2000). A specificity and targeting subunit of a human SWI/SNF family-related chromatin-remodeling complex. Molecular and Cellular Biology.

[bib31] Paakinaho V, Presman DM, Ball DA, Johnson TA, Schiltz RL, Levitt P, Mazza D, Morisaki T, Karpova TS, Hager GL (2017). Single-molecule analysis of steroid receptor and cofactor action in living cells. Nature Communications.

[bib32] Patro R, Duggal G, Love MI, Irizarry RA, Kingsford C (2017). Salmon provides fast and bias-aware quantification of transcript expression. Nature Methods.

[bib33] Quinlan AR, Hall IM (2010). BEDTools: a flexible suite of utilities for comparing genomic features. Bioinformatics.

[bib34] Ramírez F, Ryan DP, Grüning B, Bhardwaj V, Kilpert F, Richter AS, Heyne S, Dündar F, Manke T (2016). deepTools2: a next generation web server for deep-sequencing data analysis. Nucleic Acids Research.

[bib35] Rao NAS, McCalman MT, Moulos P, Francoijs K-J, Chatziioannou A, Kolisis FN, Alexis MN, Mitsiou DJ, Stunnenberg HG (2011). Coactivation of GR and NFKB alters the repertoire of their binding sites and target genes. Genome Research.

[bib36] Shain AH, Pollack JR (2013). The spectrum of SWI/SNF mutations, ubiquitous in human cancers. PLoS ONE.

[bib37] Starick SR, Ibn-Salem J, Jurk M, Hernandez C, Love MI, Chung HR, Vingron M, Thomas-Chollier M, Meijsing SH (2015). ChIP-exo signal associated with DNA-binding motifs provides insight into the genomic binding of the glucocorticoid receptor and cooperating transcription factors. Genome Research.

[bib38] Swinstead EE, Miranda TB, Paakinaho V, Baek S, Goldstein I, Hawkins M, Karpova TS, Ball D, Mazza D, Lavis LD, Grimm JB, Morisaki T, Grøntved L, Presman DM, Hager GL (2016). Steroid receptors reprogram FoxA1 occupancy through dynamic chromatin transitions. Cell.

[bib39] Takaku M, Grimm SA, Shimbo T, Perera L, Menafra R, Stunnenberg HG, Archer TK, Machida S, Kurumizaka H, Wade PA (2016). GATA3-dependent cellular reprogramming requires activation-domain dependent recruitment of a chromatin remodeler. Genome Biology.

[bib40] Trotter KW, King HA, Archer TK (2015). Glucocorticoid receptor transcriptional activation via the BRG1-Dependent recruitment of TOP2β and Ku70/86. Molecular and Cellular Biology.

[bib41] Vockley CM, D'Ippolito AM, McDowell IC, Majoros WH, Safi A, Song L, Crawford GE, Reddy TE (2016). Direct GR binding sites potentiate clusters of TF binding across the human genome. Cell.

[bib42] Wallberg AE, Neely KE, Hassan AH, Gustafsson JA, Workman JL, Wright AP (2000). Recruitment of the SWI-SNF chromatin remodeling complex as a mechanism of gene activation by the glucocorticoid receptor tau1 activation domain. Molecular and Cellular Biology.

[bib43] Wu Q, Lian JB, Stein JL, Stein GS, Nickerson JA, Imbalzano AN (2017). The BRG1 ATPase of human SWI/SNF chromatin remodeling enzymes as a driver of cancer. Epigenomics.

[bib44] Zhang Y, Liu T, Meyer CA, Eeckhoute J, Johnson DS, Bernstein BE, Nusbaum C, Myers RM, Brown M, Li W, Liu XS (2008). Model-based analysis of ChIP-Seq (MACS). Genome Biology.

